# Financial Fraud, Mental Health, and Quality of Life: A Study on the Population of the City of Madrid, Spain

**DOI:** 10.3390/ijerph16183276

**Published:** 2019-09-06

**Authors:** Encarnación Sarriá, Patricia Recio, Ana Rico, Manuel Díaz-Olalla, Belén Sanz-Barbero, Alba Ayala, María Victoria Zunzunegui

**Affiliations:** 1 Faculty of Psychology, National University for Distance Education (UNED), 28015 Madrid, Spain; 2 Joint Research Institute IMIENS, 28029 Madrid, Spain (A.R.) (B.S.-B.) (M.V.Z.); 3 National School of Public Health, Carlos III Institute of Health, 28029 Madrid, Spain; 4 Institute of Public Health, Madrid Salud, City Council of Madrid, 28029 Madrid, Spain; 5 CIBER of Epidemiology and Public Health (CIBERESP), 28029 Madrid, Spain; 6 Research Network on Health Services and Chronicity (REDISSEC), 48010 Bilbao, Spain

**Keywords:** mental health, quality of life, fraud, financial fraud, Spain

## Abstract

Over the past few decades, the financial system has engaged in abusive practices that meet the definition of fraud. Our objective is to compare the prevalence of psychological distress and levels of health-related quality of life according to having been exposed to financial fraud and its economic impact on family finances. The City of Madrid Health Survey 2017 included specific questions on exposure to financial fraud—this section was administered to half of the participants (*n* = 4425). Mental health need or caseness was defined by a score greater than two on the 12-item version of the Goldberg health questionnaire. Health-related quality of life was assessed by the Darmouth Coop Functional Health Assessment Charts/WONCA (COOP/WONCA). The prevalence of financial fraud was 10.8%. The prevalence rate ratio for caseness of those who experienced severe economic impact due to fraud was 1.62 (95%, CI 1.17–2.25; reference: no fraud), after adjustment by age, sex, social class, and immigrant status. Women experienced a decreased quality of life, even with a moderate impact of fraud, while men experienced a decreased quality of life related to fraud with severe economic impact. The current study contributes to a growing body of literature showing the effects of economic shocks on health as a result of financial fraud.

## 1. Introduction

Banking deregulation has been identified as a main cause of the recent economic crisis, and of huge public and private debts. The United States Financial Crisis Inquiry Commission concluded that “widespread failures in financial regulation and supervision proved devastating to the stability of the nation’s financial markets.… There was a systemic breakdown in accountability and ethics” [1]. The European community has recently published reports on abusive banking practices due to the miss-selling of financial products [2,3]. These practices can induce clients to buy high-risk financial products or sign abusive home mortgages, leading to personal bankruptcy or the loss of life savings, and have affected millions of people in Spain [4] through high-risk investments in products that were not designed for small savers, such as preferred shares [2,5] and abusive home loans, such as subprime mortgages, foreign currency loans, and loans with abusive clauses [3]. We will use the definition of fraud as “action that is contrary to truth and integrity, that harms those who it is committed against” [6]. Most of these abusive or fraudulent practices have been judged as scam crimes in Spain and throughout Europe.

Little research has been published on the possible effects of financial fraud on the health of the affected populations. The loss of savings due to pyramid fraud was first described in detail by Ganzini et al. [7]. That study was conducted in Oregon on elderly people who were affected by financial fraud and reported substantially worse health among those affected compared with a control group matched for age and sex [7]. Another study conducted on pensioners affected by the Maxwell scandal revealed that they experienced psychological and emotional impacts as a result of being victimized [8]. Additional studies on people affected by the Madoff financial scheme reported high levels of anxiety, depression, and health problems [9] and defined a fraud trauma syndrome characterized by severe emotional and psychological distress [10] with the following symptoms: anger, rage and pain, hopelessness and depression, anxiety, fear, nightmares, shock, numbing, emotional despair, and devastation. A recent small study in Spain reported that people who lost their savings in preferred shares fraud and people who had signed foreign exchange home loans had worse physical and mental health, and a poorer quality of life than the reference general population [11]. These studies were based on selected populations affected by fraud, and in the Spanish study comparisons were made with health indicators of a national health survey.

Using data on the adult population in Madrid, the objective of this research was to compare the prevalence of psychological distress and levels of quality of life after having been exposed to financial fraud. Our hypothesis is that financial fraud is associated with substantial mental health problems and poor health-related quality of life.

## 2. Materials and Methods

### 2.1. 2017 Madrid Health Survey

The municipality of Madrid is the most populated in Spain, with about 3 million registered inhabitants. Every four years, the municipal government of the City of Madrid (Spain) carries out a health survey of its residents aged 15 years and older. We used the survey performed in 2017 for our analysis.

### 2.2. Recruitment and Study Procedures

Participants were selected from the population of the City of Madrid via a multiple-stage random stratified sampling strategy (*n* = 8845). In the first sampling stage, homes were randomly selected from each of the 21 municipal districts of Madrid according to their population. In the second stage, random stratified sampling -involving sex and age strata according to the proportional population composition- was used to select the participants. Only one person was interviewed in each household. This person was selected as the one fulfilling the age and sex quota sampling scheme or chose at random among those who fulfilled these requirements. Information was gathered by computer-assisted telephone interviews, using the BELLVIEW CATI software. Around 87% of the population of Madrid have both landlines and cellular numbers, and 10% have exclusively cellular numbers. Thus, the sampling scheme was originally designed to recruit 50% of the sample by landline and 50% by cellular number. At the end, 57% of the total sample was interviewed by landline. The remaining participants (43%) were interviewed by cellular phone. Those participants were recruited from a list of cellular phone numbers randomly generated, until the predetermined strata quotas were completed. An error of less than 1.5% was guaranteed for the whole population in the event of relative frequency (*p* = *q* = 0.5). Overall, 83464 random calls were made to recruit 8845 participants. In 49.6% of these calls resulted in either no contact (after a maximum of 10 attempts carried out over three weeks) or no residential homes (offices, commercial firms, etc.), or numbers of people residing out of the city of Madrid; in addition, 17% of calls resulted in contacts with individuals who did not fulfill the age and sex quota for each district and lastly, 21.4% were plain refusals and about 1000 individuals started but did not complete the interview. Replacement was carried out with more calls at random to fulfill the predetermined district quota sampling scheme by age and sex.

**Figure 1:**
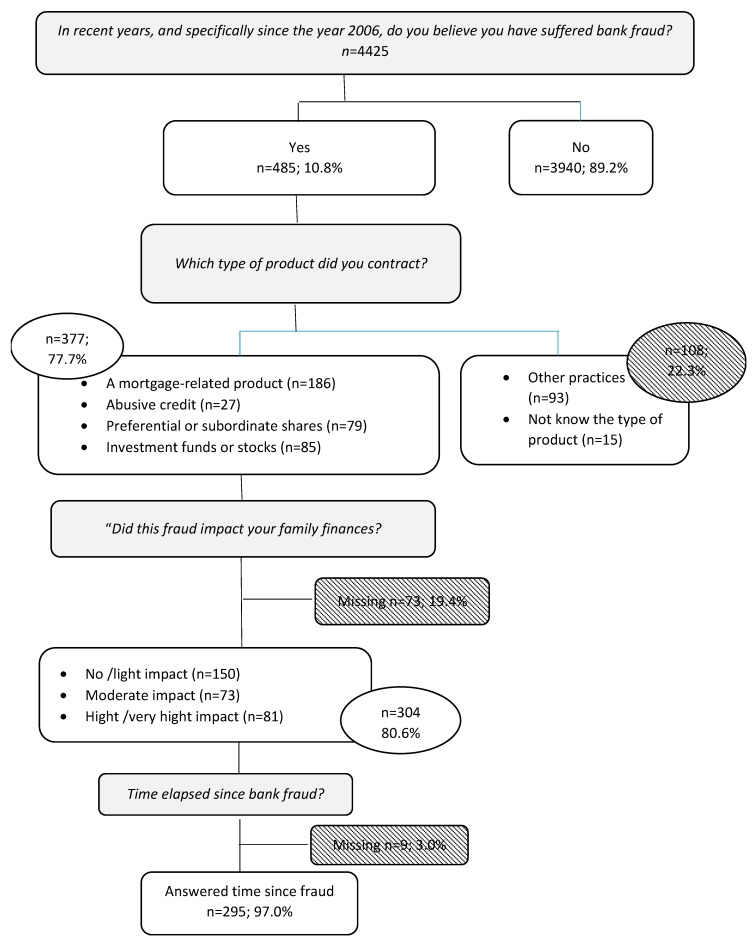
Sample definition, Madrid Health Survey 2017.

In 2017, specific questions on exposure to financial fraud were added to the core questionnaire of Madrid Health Survey, and this section was administered to half of the survey participants randomly selected (*n* = 4425), with a mean age of 48.9 years (standard deviation (SD) = 17.2, range = 15–79). Questionnaires were conducted between October and December 2017. Figure 1 is a flowchart of participants in the study according to the answers given in the fraud section. Subjects whose reported fraud type was not included in the questionnaire list (*n* = 108), or whose impact was not given (*n* = 73), were excluded from the main analysis.

### 2.3. Human Subjects

The ethics committee of the Carlos III Health Institute approved this secondary data analysis project (reference number CEI PI 51_2017_v2) dated 4 September 2017. Oral informed consent was obtained from each participant at the beginning of the telephone interview.

### 2.4. Outcomes

Mental health was assessed by the 12-item Goldberg health questionnaire (GHQ-12), a screening instrument to detect mental health needs [12]. It contains six symptoms of mental distress and six indicators of good mental health. The response categories refer to the person’s experience in the last four weeks. Each item is rated on a four-point scale: less than usual, no more than usual, rather more than usual, or much more than usual [12]. We used the scoring method proposed by Goodchild and Duncan-Jones to take into account that the response “no more than usual” to an item describing pathology reflects chronicity [13]. In the population of Spain, a GHQ-12 score greater than two has been used to indicate a high likelihood of having a common mental disorder in need of care and defines a “case” [14,15,16]. The reliability of the scale (Cronbach’s alpha = 0.86) and external validity of the measures among the Spanish population are good [17]. The total score was dichotomized with a value of 1 to indicate caseness and 0 otherwise. In this study, the Cronbach’s alpha was 0.82 and the Livingston K^2^ coefficient was 0.85.

Quality of life was assessed by the health-related the Darmouth Coop Functional Health Assessment Charts/WONCA (COOP/WONCA), which was originally proposed to assess functional health in primary care patients [18] but has been repeatedly used in the Madrid Health Survey [19]. The scale has nine items with scores ranging from one to five, for a cumulative score ranging from 9 to 45, with higher scores indicating a poorer quality of life. In the Spanish population, the COOP/WONCA test has been proven to be a reliable (Cronbach’s alpha = 0.77) and unifactorial measure of health-related quality of life (HRQOL) and useful for phone interviews [20]. In our sample, the Cronbach’s alpha was 0.77.

### 2.5. Exposure Variables

Exposure to financial fraud was defined as having contracted fraudulent financial products and assessed by the following question: “Since 2006, have you been affected by a bank fraud?”. Possible answers were yes, no, or do not know. To those who answered in the affirmative, a list of abusive bank practices was presented. The list was composed of the most frequent fraudulent financial products, including credit (abusive mortgages, foreign exchange home loans, home eviction due to unpaid mortgages, floor clauses, revolving credit) and savings (preferred shares, bank shares, and investment fund fraud), and a last option of “others”, such as those related to undue commissions on payments or excessive insurance charges or poor banking practices, which usually involve small amounts of money.

The intensity and duration of exposure to financial fraud were assessed by questions used in a previous pilot study and are similar to those used in epidemiologic research to assess dose by quantifying intensity and duration [11]. For all those who reported a type of financial fraud included in the list of credit or savings types, two additional questions were asked. Intensity was assessed by a single question on the economic impact of the fraud on the family finances, answered in five categories: no impact, light, moderate, high, very high. For the analysis, a combined variable was created with four categories: no fraud, fraud with no/light impact, moderate, and severe impact. The duration of exposure was assessed by the number of months between the date the person was made aware of the fraud and the date of the interview.

To further describe the judicial process following the awareness of fraud, questions on claims and economic compensation were asked. Claims were categorized as none, judiciary, or extrajudiciary. Economic compensation was asked in terms of none, partial, or total.

### 2.6. Covariates

Individual characteristics (age, sex, social class, and immigrant status) were included as potential confounders of the association between mental health and financial fraud if they were related to mental distress and quality of life and internet scams, economic shocks, or mortgages, since there is scant literature on financial fraud and mental health. Date of birth was self-reported and age was calculated from it. Occupational social class was classified into three categories based on the occupation of the main provider of the household: low (e.g., manual workers), middle (e.g., office workers and intermediate occupations), and high (e.g., managers and university professionals), using the National Classification of Occupations [21]. Immigrant status was assessed by place of birth as Spain or another country.

### 2.7. Statistical Analysis

Population characteristics were compared across categories of financial fraud. Bivariate analyses were conducted to examine associations between caseness and intensity of financial fraud by chi square test of linear trends. ANOVA was used to compare means of health-related quality of life by the impact of the financial fraud on the family economy using the F-test for linearity. Tests of homogeneity were used for the associations of claims and economic compensation.

Multiple Poisson regression with robust variance was used to estimate the prevalence ratio of caseness (GHQ ≥ 3) and multiple linear regressions for the COOP/WONCA scores according to levels of economic impact of the financial fraud, adjusting for age, sex, social class, and immigrant status. Multiplicative tests for interaction were included in both models to examine whether there were gender differences in the effects of economic fraud on mental health and quality of life. Data were analyzed with Stata, version 14 (StataCorp, College Station, TX, USA)). All analyses were carried out using weighted coefficients. All frequencies shown in the text and tables are unweighted absolute frequencies and weighted relative frequencies.

## 3. Results

**Figure 2:**
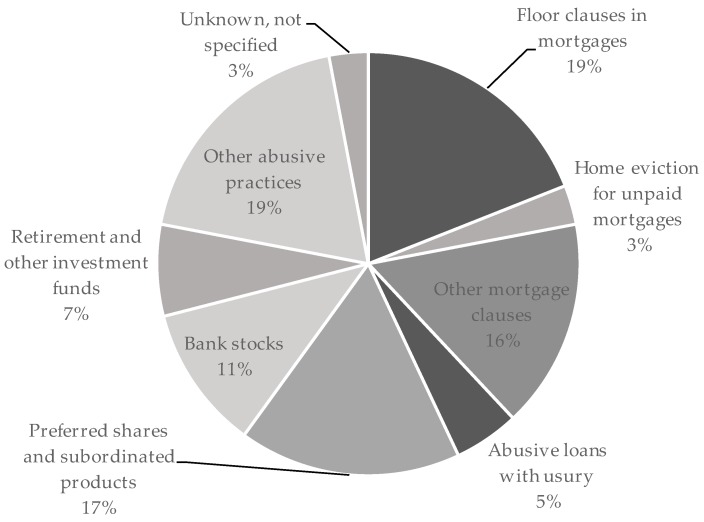
Distribution of financial fraud types as reported in the 2017 Madrid Health Survey (*n* = 485).

Figure 2 shows the distribution of financial fraud as reported in the 2017 Madrid Health Survey. Among the participants, 10.8% (*n* = 485/4425) reported to have been affected by financial fraud. The majority of contracted products were banking products, although some participants reported nonbank financial fraud (< 8%). Savings losses were related to preferred shares (16.9%, *n* = 82), other fraudulent stocks (10.6%, *n* = 51), and retirement and other investment funds (7%, *n* = 34); among those who had contracted a mortgage-related product, 18.7% (*n* = 91) had been affected by abusive floor clauses, 16.4% (*n* = 80) reported other kinds of abusive mortgages including foreign exchange mortgages, and 3.1% (*n* = 15) reported home evictions due to unpaid mortgages; and finally, 5.5% (*n* = 27) reported abusive loans or loans with usury. There were 19.1% (*n* = 93) who reported other abusive practices, mostly undue bank commissions, and an additional 3% (*n* = 13) who did not know or did not remember further details of the reported financial fraud after having answered “yes” to the opening question.

The mean age of the participants who reported fraud was 50.8 years (standard deviation (SD) = 14.9, range = 16–79), 49.6% were women, 39.3% declared occupations indicative of high social class, 26.9% reported occupations classified as middle social class, and 33.8% reported manual occupations classified as low social class, and 15.9% were born outside of Spain. Regarding those who did not report financial fraud, their mean age was 48.7 years (standard deviation (SD) = 17.4, range = 15–79), 46.0% were women, 38.9% declared occupations indicative of high social class, 22.6% reported occupations classified as middle social class, and 38.5% reported manual occupations classified as low social class, and 18.5% were born outside of Spain.

Descriptive results of prevalence of mental health problems (GHQ-12) and mean health related quality of life (COOP/WONCA) for the total sample stratified by age, gender, social class and immigrant status are shown in Table 1. The prevalence of mental health problems was lower in men (*p* < 0.001) and those with high social class (*p* < 0.001). The health-related quality of life was lower in women (*p* < 0.001) and decreased with decreasing social class (*p* < 0.001) and increasing age (*p* < 0.001).

Table 2 shows the distribution of those who reported any of the types of fraud included in the questionnaire list (*n* = 304), excluding other types of fraud and those who did not remember what kind of fraud, according to its economic impact on family finances and the time elapsed since the subject was aware of the fraud. The mean time elapsed since the awareness of fraud was longer than five years for those with moderate or severe impact, while those with less impact tended to report shorter times, around four years.

COOP/WONCA had a normal distribution with a mean of 20.4 and standard deviation of 5.4. Table 3 shows the prevalence of caseness and the average health-related quality of life (COOP/WONCA mean and standard deviation) according to the economic impact on family finances. The prevalence of caseness increased (*p* = 0.016) and quality of life decreased (*p* < 0.001) with the impact of fraud. Less than one-third decided not to submit a claim for compensation—of those who started a claim, half started a judiciary process, and half tried an extrajudiciary settlement. Of those who made a claim for compensation, half got none—among the other half, 40% had partial compensation and 60% total compensation. No significant associations were found between the type of claim or compensation and mental health or quality of life.

Table 4 shows the distribution of COOP/WONCA scores by the economic impact on family finances. Generally, quality of life worsened (COOP/WONCA scores increased) with increased economic impact of the fraud. More specifically, being disturbed by negative feelings, having difficulty in daily life, reporting limitations in social activities due to health, feeling chronic pain, perceiving less availability of help, and having worse overall quality of life were significantly associated with economic impact of fraud; there was a nonsignificant trend of worse physical fitness and self-rated overall health with increasing economic impact of fraud (*p* = 0.08 and *p* = 0.07, respectively). Changes in self-rated health during the last two weeks were not related to the severity of the economic impact of fraud (*p* = 0.367). This was expected, given that almost all the fraud had happened more than a year ago and changes in health in the last two weeks are not likely to be related to events that happened in a much wider time window.

Table 5 shows the unadjusted and adjusted prevalence rate ratios (PRRs) of caseness by the economic impact of fraud on family finances. Prevalence rate ratios changed little after adjusting by age, sex, social class, and immigrations status. Those reporting severe economic impact had a significantly increased prevalence of caseness in both the unadjusted (PRR: 1.52; 95% CI 1.09–2.12) and adjusted (PRR: 1.62; 95% CI 1.17–2.25) models. Among those reporting a moderate impact, while they also had increased prevalence compared to those with no fraud, the confidence interval of the prevalence ratio of caseness includes unity (95% CI 0.97–2.07). Lastly, the prevalence of caseness among those to report fraud with no or light economic impact was similar to the prevalence among those who had not experienced any financial fraud.

The multiplicative term to test for interactions between sex and economic impact was not significant, indicating that we cannot discard the hypothesis that the association between the economic impact of financial fraud and caseness had similar strength for men and women. A sex-stratified analysis is shown for informative purposes.

**Figure 3:**
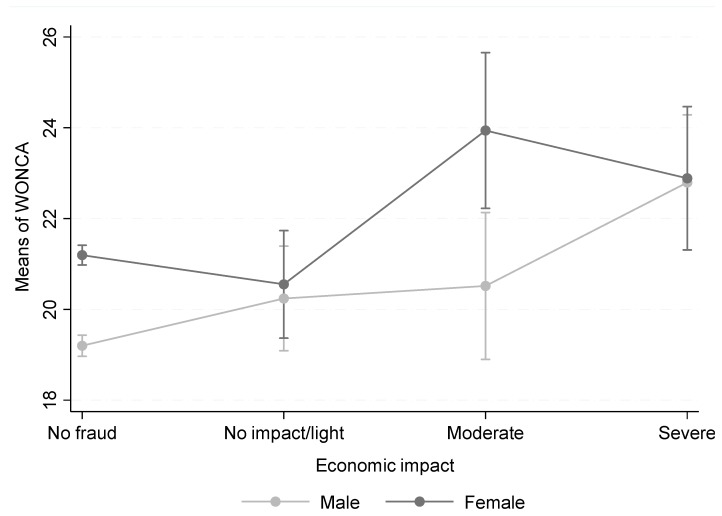
COOP/WONCA scores by economic impact on family finances Higher COOP/WONCA scores mean lower health-related quality of life. The *p*-value for interaction of sex and economic impact on family finances = 0.042.

Figure 3 shows the results of adjusted multiple linear regression to examine the association between health-related quality of life and the economic impact of financial fraud, adjusting by age, social class, and immigrant status. The figure shows that, as the economic impact on family finances increases, quality of life decreases for both men and women. The test for interaction between gender and economic impact on quality of life was statistically significant (*p* = 0.042). In fact, women experienced noticeably decreased quality of life even with a moderate impact of fraud, while the effect on men’s quality of life was restricted to fraud with a severe economic impact on family finances.

## 4. Discussion

The results of this study on a representative sample of residents of Madrid support the hypothesis that those who have been exposed to financial fraud have greater mental health needs and poorer health-related quality of life than those who have not. In addition, a gradient was observed: the probability of having mental health needs (caseness) increased and quality of life decreased as the economic impact of fraud on family finances increased. Furthermore, among women, the impact of financial fraud on health-related quality of life was already detectable at moderate levels of fraud, while among men these effects were only significant for severe fraud. The vulnerability of women’s health to economic shocks has also been reported in previous studies. Vasquez-Vera et al. [22] reported that for women the threat of eviction is enough to affect them, while men only are affected by the eviction itself.

The above findings suggest that citizens who have suffered a loss of savings due to the miss-selling of financial products, and those who are in debt because of bank fraud related to abusive mortgages or credit loans, face mental health problems and decrements in their quality of life beyond their economic losses. These results are consistent with previous research about victims of financial fraud [8,9,10,11,23]. These findings open a field of inquiry on the potential causal health consequences of financial fraud.

Due to the limited research on the health consequences of financial fraud, we interpret our findings in light of three topics in the literature: the emotional impact of internet fraud with financial loss [24], the effects of indebtedness on mental health and quality of life [25], and the health effects of foreclosure and eviction [22,26,27]. These studies could shed light on the effects of financial fraud on health, since they have in common economic loss due to fraud, meaning people have been misled, with a breach of confidence and asymmetry of information. Financial fraud shares these characteristics; it is the result of dishonest practices leading to economic loss because of deceit.

Research about internet fraud with financial loss shows the emotional impact of fraud [24,28,29,30,31]. Victims described the fraud as “devastating” and “soul-destroying”. They described their emotional responses following the fraud as distress, sadness, anger, stress, worry, shock, loneliness, and shame that appeared to stem from self-blame. Some participants were so devastated that they had thoughts of suicide [24]. A quantitative analysis across internet fraud categories showed that participants reported the highest emotional impact for types of fraud that supposed a relationship between victims and offenders (e.g., romance scams and advanced fee fraud), adding eventual betrayal to the deception and financial loss [28].

The economic losses caused by financial fraud can be associated with debt or cause it. Indebtedness has been described as one of the strongest risk factors for mental health, in both older adults [32] and other populations [25,33]. Debt can be a source of significant distress [34,35] and strongly associated with suicidal thoughts [36]. We argue that being in a state of indebtedness with a bank, indebtedness resulting from fraud that the bank itself has committed toward a client, and abusing trust and power and using fraudulent information will harm mental health beyond what could be expected from indebtedness accepted in an honest deal. Abusive practice of the banking sector in Spain during the last 15 years has been contrary to European legislation and the subject of litigation in European courts. Frequently, bank transactions with clients who are small savers or ask for home loans occur in the context of a long-term relationship, during which the client has used the bank to deposit his/her salary or conduct everyday business according to the advice of local bank employees. In addition to the economic loss, negative feelings of deception, guilt, shame, insecurity, and helplessness arise due to these breaches of trust [37].

At the extreme, home foreclosures were often the consequence of subprime mortgages granted by banks to people with insecure jobs or unstable incomes. According to a systematic review of 47 studies, individuals affected by foreclosure reported a higher frequency of depression, anxiety, psychological distress, and suicide compared with the general population or people who did not have these experiences. Foreclosure has an impact on the health and well-being of all members of the family, particularly children [38]. Examining possible pathways between home foreclosure and mental health, Vasquez-Vera [22] proposed that housing insecurity acts as a stressor that increases the incidence of major depression and generalized anxiety disorder, as demonstrated in the United States [39]. A systematic review by Downing proposed four distinct pathways at both the individual and community level (stress, effect budgeting, frustration-aggression, and trust) mediating the effects between the societal forces that lead to foreclosure and the individual’s health [26]. In both reviews, mental health needs were identified in the affected populations; in particular, increased frequency of depression, anxiety, and suicide. In Spain, poor mental health was more likely to occur among people who were in the foreclosure process (from being in default to being evicted) than those who were up to date on their payments or had no mortgage [37,40]. In recent years, in Spain, as a consequence of the economic crisis and the abusive behaviour of banks, many families have been unable to pay for utilities, healthy foods, or adequate dwellings [37,40,41].

The relationship between financial fraud, victimization, and mental health is supported by research identifying financial loss, and more specifically financial loss because of fraud, as a stressor associated with negative emotions and by research linking stress with depression, anxiety, and other mental illness [42,43]. It is possible that negative emotions act as a mediator in the relationship between fraud and mental health, which should be assessed in future research.

### Strengths and Limitations

The originality of the research questions, the representativeness of the sample, and the use of a questionnaire that was validated by the results of a pilot study should be considered as strengths of the current study. The main limitation of this research is its cross-sectional nature, since mental health, quality of life, and financial fraud were assessed in the same survey, but we ascertained that all fraud had taken place at least four months before the interview, while the GHQ refers to the previous four weeks and WONCA to the last two weeks. To avoid a potential response bias, questions on financial fraud were formulated at the end of the interview, once all questions on health status had been asked. Associations could also be explained by reverse causality if those with poor mental health were particularly vulnerable to abusive banking behaviour; recent research has linked emotional arousal with vulnerability to financial fraud due to false advertising, even more so in older adults [44].

Limitations in sample size hinder our ability to stratify the study population by type of financial fraud. Thus, we were unable to separately analyze those affected by credit or savings fraud. As stated above, these two populations are demographically and economically different. Credit fraud was addressed with regard to those who were searching for credit to buy a home, start a small business, or pay previous debts. Savings fraud was directed to people who were looking for investments to obtain some interest on their life savings. The combined analysis of these two subpopulations may have attenuated the association between fraud and health, and, even more importantly, we were unable to examine the special vulnerability of some groups: women, heads of single-parent families, older adults, immigrants, and those with previous mental illness. In fact, previous research in Spain reported that anxiety crises exacerbate or become more frequent after people are affected by bank fraud [11] or the foreclosure process [41]. We also have to recognize as a limitation that relevant variables related to mental health such as chronic diseases, other stressful events, marital status or social support have not been evaluated in their possible roles as mediating or modifying factors in the relationship between financial fraud and mental health. Although individual personal characteristics and social or health history are likely to increase vulnerability, we argue that beyond those individual characteristics, abusive banking practices are the public health factor under investigation, and that these practices should and can be stopped through greater regulation and supervision of the financial sector.

Lastly, the procedure to collect the information, a telephone interview, could be a deterrent to answering the questions in detail, as suggested by the smaller number of people who gave exact information on dates of claims and economic compensation. Briefly, a considerable number of subjects who were willing to answer the opening question on having been affected by financial fraud, and recognized the type of fraud as one on the questionnaire list, declined to answer the questions on dates and types of claims and economic compensation. Some of them stated that they did not remember those details, while others stated that they preferred not to answer those questions. Research should be undertaken to assess how the type of claim (judiciary or extrajudiciary) and type of compensation (none, partial, or total) moderate the mental health effects and the effects of fraud on quality of life.

## 5. Conclusions

Even though the psychological and biological mechanisms by which financial fraud acts on mental health have not yet been explored, the current study contributes to a growing body of literature showing the effects of economic shocks on health that are the result of abusive bank practices, including the mis-selling of investment products and mortgages. Future studies should aim to determine whether the claims and compensation trajectories of financial fraud victims moderate the long-term impact of financial fraud on health. If the findings of this study are replicated by similar studies in the European region, financial fraud should be considered a new determinant of population health, financial institutions including banks should be held accountable for these abusive practices, and victims should receive adequate mental health care and be compensated not only for the economic losses, but also for the damage to their mental health and quality of life.

## Figures and Tables

**Table 1:** Prevalence of caseness (12-item Goldberg health questionnaire; GHQ-12) and average quality of life (Darmouth Coop Functional Health Assessment Charts/WONCA; COOP/WONCA) according to age, gender, social class and immigrant status.

Variable	Unweighted *n*	Mental Health Problems (GHQ-12)		Quality of Life (COOP/WONCA)	
		Weighted %	*p*-Value	Mean (SD)	*p*-Value
Age (years)					
15–29	750	22.8	0.054 ^a^	18.4 (4.9)	0.000 ^a^
30–44	1198	21.1		19.7 (5.2)	
45–64	1436	23.0		21.0 (5.6)	
>65	1041	18.0		21.9 (5.2)	
Gender					
Men	2035	16.8	0.000 ^b^	19.2 (4.8)	0.000 ^b^
Women	2390	25.1		21.4 (5.7)	
Social class					
High	1743	17.8	0.000 ^a^	19.1 (4.8)	0.000 ^a^
Middle	1009	22.1		20.7 (5.4)	
Low	1584	23.9		21.5 (5.6)	
Immigration status (Place of birth)					
Outside of Spain	764	23.2	0.166 ^b^	20.4 (5.1)	0.942 ^b^
Spain	3661	20.8		20.4 (5.5)	

Note. Higher COOP/WONCA scores mean lower health-related quality of life. ^a^ Chi-square for linear trends or ANOVA for linearity. ^b^ Chi-square for homogeneity or ANOVA for equal means.

**Table 2:** Economic impact of fraud on family finances.

Economic Impact of Fraud	Unweighted *n* (weighted %) ^a^	Time since Awareness of Fraud (Months)
		(Mean, SD)
No fraud (4425 − 485 = 3940)	3940 (92.9)	
No or light impact	150 (3.5)	49.4 (35.1)
Moderate	73 (1.7)	66.6 (39.8)
Severe or very severe	81 (2.0)	68.8 (39.5)

Note. ^a^ Excluded from the table are 181 subjects whose reported fraud type was not included in the questionnaire list (*n* = 108) or whose impact was not given (*n* = 73).

**Table 3:** Prevalence of caseness (GHQ-12) and average quality of life (COOP/WONCA) according to financial fraud history.

Variable	Unweighted *n*	Mental Health Problems (GHQ-12)		Quality of Life (WONCA)	
		Weighted %	*p*-Value	Mean (SD)	*p*-Value
**Economic impact**			0.016 ^a^		<0.001 ^a^
No fraud	3940	20.4		20.3 (5.4)	
No/light impact	150	20.6		20.4 (5.4)	
Moderate	73	28.8		22.4 (5.4)	
Severe/very severe	81	31.2		23.2 (5.4)	
**Claims**			0.884 ^b^		0.877 ^b^
None	87	22.1		21.6 (5.5)	
Judiciary	106	26.6		21.8 (5.5)	
Extrajudiciary	109	27.6		21.7 (5.5)	
**Economic compensation**			0.896 ^b^		0.414 ^b^
None	102	26.9		21.3 (6.1)	
Partial	42	28.5		22.6 (6.1)	
Total	63	28.4		22.0 (6.1)	
In process	6	18.3		22.4 (6.1)	

Note. ^a^ Chi-square for linear trends or ANOVA for linearity. ^b^ Chi-square for homogeneity or ANOVA for equal means.

**Table 4:** WONCA items according to economic impact on family finances.

Items’ Contents	Unweighted *n*	Economic Impact on Family Finances (Weighted %)				*p*-Value ^a^
		No Fraud	No or Light Impact	Moderate	Severe or Very Severe	
Physical fitness activity						0.077
Very intense	973	23.5	24.6	15.7	10.3	
Intense	580	13.7	13.5	12.8	7.5	
Moderate	1872	43.6	46.1	46.3	49.7	
Light	629	14.9	12	18	26.4	
Very light	190	4.3	3.8	7.1	6.1	
Disturbed by negative feelings						0.044
None	2329	55.4	53.1	50.9	49	
Some	964	22.4	29.9	23.9	16.6	
Moderately	519	12.3	7.5	10.2	17.4	
A lot	301	6.7	9.2	8.4	11.2	
Very much	131	3.3	0.2	6.5	5.8	
Daily activities difficulties						<0.001
None	2875	68.6	57.5	59	53.5	
A little bit	668	15.5	22.3	13.3	14.2	
Moderately difficult	492	11.3	14.3	14.3	21.3	
Highly difficulty	160	3.5	5.3	12.1	9.2	
Completely unable	49	1.2	0.7	1.3	1.9	
Social activities limited by health						0.001
None	3345	79.4	73	66.6	67.5	
A little bit	435	10	17.3	7.3	16.5	
Moderately	260	6	7.4	12.3	10.2	
A lot	137	3.1	1.6	7.4	3.9	
Completely	67	1.6	0.7	6.3	1.9	
Health status change (last two weeks)						0.367
Much better	336	8.1	7	3.8	11.7	
Somewhat better	464	11.4	10.5	7.4	8.1	
Same	3155	73.8	73.5	77.2	69.9	
Somewhat worse	242	5.5	8.7	8.4	8.4	
Much worse	47	1.1	0.3	3.2	1.9	
Self-rated health (today)						0.07
Excellent	465	11.2	9.1	9.3	3.9	
Very good	842	20.1	18	14.6	15.7	
Good	1994	46.7	50.6	44	42.2	
Fair	784	18.4	19.2	23.9	33	
Poor	159	3.7	3.1	8.1	5.2	
Pain						0.001
None	1699	40.2	43.7	35.2	19.4	
Very light pain	937	22.3	16.2	13.3	27.9	
Light pain	548	12.5	16.8	8.4	14.5	
Moderate pain	735	17.1	17.3	31.1	31	
Severe pain	325	7.9	6	12	7.1	
Availability of help						0.013
Yes, everyone	707	16.3	21.2	17.8	1.9	
Yes, a lot of people	1300	30.5	21.2	26.5	29.9	
Yes, some people	1044	24.9	28.5	29.7	24.4	
Yes, a few people	1007	23.7	25.6	20.5	39.2	
Nobody	186	4.6	3.6	5.5	4.6	
General quality of life						<0.001
Very good, could not be better	499	11.9	11.2	13.2	6.4	
Good	2146	51	46.0	35,7	34,9	
Fair	1430	33.2	39.8	38.5	51.4	
Bad	130	2.9	2.6	12.6	5.5	
Very bad, could not be worse	39	1	0	0	1.9	

Note. ^a^ Chi-square for linear trends.

**Table 5:** Prevalence rates ratio of mental health problems by economic impact.

Economic Impact (Ref: No Fraud)	Unadjusted Prevalence Rates (CI 95%)			Adjusted Prevalence Rates ^1^ (CI 95%)		
	Both genders	Male	Female	Both genders	Male	Female
No/light impact	1.01 (0.72–1.41)	1.09 (0.64–1.86)	0.99 (0.64–1.53)	1.06 (0.75–1.49)	1.15 (0.67–1.95)	1.01 (0.64–1.59)
Moderate	1.41 (0.96–2.06)	1.26 (0.66–2.38)	1.61 (1.02–2.52)	1.41 (0.97–2.07)	1.22 (0.61–2.43)	1.64 (1.06–2.53) *
Severe/very severe	1.52 (1.09–2.12) *	1.55 (0.91–2.64) *	1.58 (1.04–2.38) *	1.62 (1.17–2.25) *	1.64 (0.96–2.79) *	1.64 (1.09–2.47) *

Note. ^1^ Adjusted by gender, age, social class, and immigration. * Significant at 5% level. CI 95%, confidence interval at 95%.
